# Powered air-purifying respirators do not compromise air quality in the operating theatre

**DOI:** 10.1016/j.infpip.2021.100140

**Published:** 2021-04-15

**Authors:** Deirdre Brady, Nicola Boran, Dara Ann O'Malley, Jessy Joy, Aoife O'Neill, Jeffrey Dalli, Ronan Cahill, Jincy Jerry

**Affiliations:** Mater Misericordiae University Hospital, Dublin, Ireland

**Keywords:** PAPR, Contamination, Operating theatre, Settle plates, COVID-19

Dear Editor,

The impact of COVID-19 on normal operations in healthcare has been immense. With the urgent requirement for increased bed capacity to care for larger numbers of acute medical patients, many institutions suspended some or all elective surgery during COVID surges. The Mater Misericordiae University Hospital is a city centre acute hospital and a national referral centre for respiratory intensive care. In the past year, more than one thousand patients with COVID-19 have been admitted to the hospital. This service must be balanced against referral services including emergency and specialist time-dependent surgery e.g. cancer surgery, transplants. Operating during a pandemic introduces concerns about spread of COVID-19 within the operating theatre and has led surgical colleagues to consider the use of a higher level of respiratory protection, in the form of powered air-purifying respirators (PAPR). Dalli *et al.* set out to evaluate the use of PAPR in general surgery, firstly for user satisfaction [1] during a simulation exercise and secondly, as a clinical trial [2].

This use of PAPR in theatre was discussed with the infection prevention and control team as is the norm with any new personal protective equipment being brought in to use at our institution. Due to published evidence of the effect of door opening on theatre air flow [3], concern was raised about the impact of a powered device having such an effect and the potential increase in contamination of the operative field or instruments leading to a consequential rise in surgical site infection cases. With a proposal to start the clinical trial on patients undergoing gastrointestinal surgery, we decided to perform microbiological sampling with settle plates to investigate whether use of PAPR reduced the safety of surgery by increasing contamination.

The theatre complex at MMUH is relatively modern, being newly opened less than 10 years. Settle plate testing was carried out in a conventionally ventilated operating theatre (>/= 25 air changes per hour) under three conditions; an empty theatre for baseline measurement, a surgical simulation with the operators donning usual surgical attire with a surgical mask and no PAPR and finally a surgical simulation with the operators using PAPR. 9cm diameter Columbia blood agar plates were positioned at ten points in the theatre representing the positions of five patient body regions (head, thorax, abdomen, hip and leg), the position of the instrument trolley and other positions at the edge of the surgical space and the theatre itself, and were collected after one hour. Plates were incubated in air at 37°C for 48 hours. Colony counts were assessed and the results are presented in Table I.

**Table I:** Results of settle plate testing presented in absolute colony forming units per 9cm agar plate

Position	Empty theatre	In-use theatre, normal surgical attire; bacterial colony forming units (cfu) per plate	In-use theatre, PAPR worn by operative team; cfu per plate
1 Operating table	No growth	4	2
2 Operating table	No growth	4	5
3 Operating table	No growth	2	6
4 Operating table	No growth	1	2
5 Operating table	No growth	1	1
6 Instrument trolley	No growth	1	4
7 1M away/1M height/anaesthetic side	No growth	1	4
8 1M away/1M height/scrub side	No growth	3	2
9	No growth	2	4
10	No growth	3	8

Settle plate testing has largely been replaced by active air sampling methods for evaluation of theatre air quality e.g. during theatre commissioning. We chose settle plate testing for this study to give us a more realistic picture of the particles actually landing in the operative field and on the instrument trolley. Other plate positions were in line with standard theatre air sampling following the 1m out from the wall and 1m up from the floor rule.

Statistical analysis has previously confirmed that passive and active monitoring correlate in a comparable way with the quality of air in operating theatres [4]. UK guidance recommends threshold values of not more than 10 CFU/m3 using active sampling in an empty operating suite or <180 CFU per/m3 in operation [5]. There is no current UK or Irish recommendation for passive sampling but thresholds have been included in guidance in other jurisdictions, a summary of which was provided by Pasquarella *et al.*, in 2019 [6]. In our study, testing in an empty theatre confirmed no baseline level of contamination. Thus, contamination found in surgical simulations is attributable to the activity undertaken. Whether the operating theatre team used standard surgical attire with face masks or PAPR, there was little difference between the levels of contamination and all was within acceptable limits quoted in the available international guidance. This allowed the clinical trial to proceed.

The Infection Prevention and Control Team can assist with the introduction of new technology while maintaining safe theatre standards.

## Conflict of interest statement

Prof Cahill is named on a patent filed in relation to processes for visual determination of tissue biology, receives speaker fees from Stryker Corp, research funding from Intuitive Corp and holds research funding from the Irish Government in collaboration with IBM Research in Ireland. Dr Dalli is employed as a researcher on the same collaboration.

## Ethics statement

Approval by research ethics committee was not required as this study was environmental only and did not involve patients.

## Funding

No funding was sought for the researching and writing of this letter.
